# Concordant and opposing effects of climate and land-use change on avian assemblages in California’s most transformed landscapes

**DOI:** 10.1126/sciadv.abn0250

**Published:** 2023-02-22

**Authors:** Steven R. Beissinger, Sarah A. MacLean, Kelly J. Iknayan, Perry de Valpine

**Affiliations:** ^1^ Department of Environmental Science, Policy and Management, University of California, Berkeley, Berkeley, CA, USA.; ^2^ Museum of Vertebrate Zoology, University of California, Berkeley, Berkeley, CA, USA.; ^3^ Department of Biology, University of La Verne, La Verne, CA, USA.; ^4^ San Francisco Estuary Institute, Richmond, CA, USA.

## Abstract

Climate and land-use change could exhibit concordant effects that favor or disfavor the same species, which would amplify their impacts, or species may respond to each threat in a divergent manner, causing opposing effects that moderate their impacts in isolation. We used early 20th century surveys of birds conducted by Joseph Grinnell paired with modern resurveys and land-use change reconstructed from historic maps to examine avian change in Los Angeles and California’s Central Valley (and their surrounding foothills). Occupancy and species richness declined greatly in Los Angeles from urbanization, strong warming (+1.8°C), and drying (−77.2 millimeters) but remained stable in the Central Valley, despite large-scale agricultural development, average warming (+0.9°C), and increased precipitation (+11.2 millimeters). While climate was the main driver of species distributions a century ago, the combined impacts of land-use and climate change drove temporal changes in occupancy, with similar numbers of species experiencing concordant and opposing effects.

## Teaser

Climate and land-use changes caused birds to decline in urban Los Angeles but not in the agricultural Central Valley.

## INTRODUCTION

Climate and land-use change are the greatest threats to biodiversity (*1*, *2*). While climate is a dominant driver of species distributions (*3*–*5*) and climate change is expected to threaten one-sixth of the world’s species through extirpation of populations (*1*, *6*), land-use change has endangered more species to date (*2*). How these threats influence assemblages of species, however, depends on the cotolerance of species to these stressors (*7*, *8*) and the particular combination of climate and land-use change that occurs at a location. The two threats could cause concordant effects that favor or disfavor the same species (*9*, *10*), which would result in “windfalls” and “double-whammies,” respectively, for species. Concordant effects amplify impacts and lead to more rapid changes than expected if considering each threat alone. Alternatively, species could respond to each threat in a divergent manner; in that case, climate change could exert a positive influence and land-use change a negative effect, or vice versa. This should result in opposing effects that moderate the impacts of climate and land-use change in isolation (*11*). What causes species to exhibit concordant or opposing responses and, as a result, the net effects of climate and land-use change on contemporary assemblages depend on the degree that species are exposed to each threat, their sensitivity to threats, and their adaptive capacity to respond (*12*–*14*).

Concordant and opposing effects may also result from different dimensions of climate and land-use change. Temperature and precipitation are the most frequent dimensions of climate change studied. Depending on the direction and magnitude of climate change and whether species’ niches track changes in precipitation and temperature, both or neither, opposing (*15*, *16*) and concordant (*17*) effects on range shifts and occupancy have occurred. Urbanization and agriculture, which represent the dominant land-use changes over the past century (*18*, *19*), usually reduce species richness and phylogenetic diversity and favor generalists and non-native species (*10*, *20*–*23*). Thus, species could exhibit concordant sensitivity to urbanization and agriculture, and long-term exposure to these threats should lead to homogenization of assemblages (*24*). However, some impacts of these land uses differ and could result in opposing effects on species. Agricultural expansion and intensification often replace natural land cover with crop monocultures and pastures, resulting in the potential for elevated mortality from pesticides and other types of pollution (*25*, *26*). Urbanization destroys natural land cover and increases mortality risk due to predation by domestic cats and collisions with automobiles and building windows (*27*). However, the addition of buildings and non-native plant species to urban areas increases nesting sites and food resources for animals tolerant of human activity (*28*–*30*).

Here, we examine how heterogeneous patterns of climate and land-use change have affected avian occupancy and diversity over the past century in adjacent regions with extensive but contrasting histories of land-use and climate change. We reconstructed an early 20th century ecological baseline using unique historical resources: (i) systematic bird surveys conducted by Joseph Grinnell from 1895 to 1904 at 71 sites around the Greater Los Angeles metropolitan area where he grew up and in the surrounding foothills (hereafter “Los Angeles”) and in California’s Central Valley and surrounding foothills (hereafter “Central Valley”) with colleagues in the early 1900s and (ii) hand-digitized land-use maps from the same period. These were matched with contemporary bird resurveys and measures of land-use and climate change at the same sites (fig. S1). Los Angeles and the Central Valley share the same avian species pool but have experienced disparate changes in climate (*31*) and have been differently transformed by land-use change.

We first quantify how avian occurrence and diversity in Los Angeles and the Central Valley have changed over the past century using occupancy models to account for differences in detection among species and over time. Next, we compare the effects of climate and land-use change on species persistence and colonization, and we develop a novel measure that uses the derivative of occupancy change to quantify the sensitivity of species to each threat. Last, we use counterfactuals to estimate the regional impacts on species from each threat independently to examine the frequency of concordant and opposing effects. Our approach directly quantifies the impacts of climate and land-use change on diversity in contrast to indirect inference using a space-for-time substitution used by most studies (*21*). The long lapse between surveys reduces the potential for delayed effects and transient dynamics, which can take a century to unfold (*32*, *33*).

## RESULTS

### Exposure to climate and land-use change over the past century

**Fig. 1.**
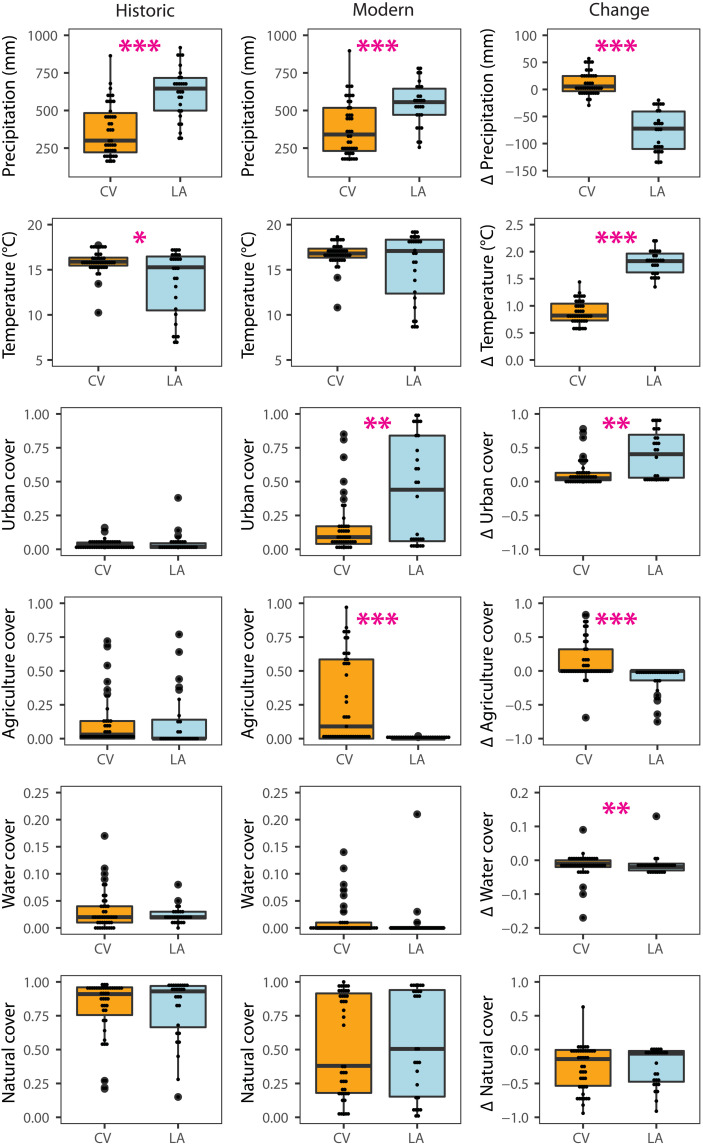
Regional differences in exposure to climate and land-use change over the past century for survey sites in the Central Valley (CV) and Los Angeles (LA). (**Left**) Mean site-level historic values at the beginning of the 20th century. (**Middle**) Modern site-level values. (**Right**) Change over the past century. Annual total precipitation and mean annual temperature were calculated over 30-year periods corresponding to the historic (1900–1929) and modern (1988–2017) surveys. Land-use measures for survey sites were made at a 1-km scale. Differences between regions within time periods from Mann-Whitney *U* tests are marginally significant (*P < 0.1), significant (**P < 0.05), and highly significant (***P < 0.001).

The Central Valley and Los Angeles sites experienced divergent trends in climate and land-use change over the past century (Fig. 1 and table S1). In the early 20th century, sites in the Central Valley were marginally warmer and significantly drier than sites in Los Angeles. While both regions warmed over the past century, strong warming (+1.8°C) and drying (−77.2 mm) characterized sites in Los Angeles, while moderate warming (+0.9°C) and wetting (+11.2 mm) occurred at Central Valley sites. Today, sites in the Central Valley and Los Angeles still differ climatically but less so than a century ago (table S1).

Early 20th century sites in Los Angeles and the Central Valley were primarily undeveloped, and natural land cover comprised 81% of both regions (Fig. 1 and table S1). Historically, the regions did not differ in the percent of land use by urbanization, agriculture, surface water, or natural land cover. Over the past century, however, urbanization increased by 7.8 times in Los Angeles to dominate land use in the region, while agriculture and surface water were nearly eliminated. In contrast, the Central Valley sites experienced a 2.5-fold expansion of agriculture and a 1.5-fold increase in urban cover. Natural cover remains at sites in both regions at a similar level. Within each region, modern land use for all categories differed significantly from its historic coverage except for water cover (Fig. 1 and table S1).

### Changes in avian occupancy and diversity

A diverse set of bird species were detected in Los Angeles (*n* = 141) and the Central Valley (*n* = 137) throughout this study, and species composition of the two regions was very similar. Of the 148 species analyzed, 7 (4.7%) were detected only in the Central Valley and 11 (7.4%) only in Los Angeles (table S2).

**Fig. 2.**
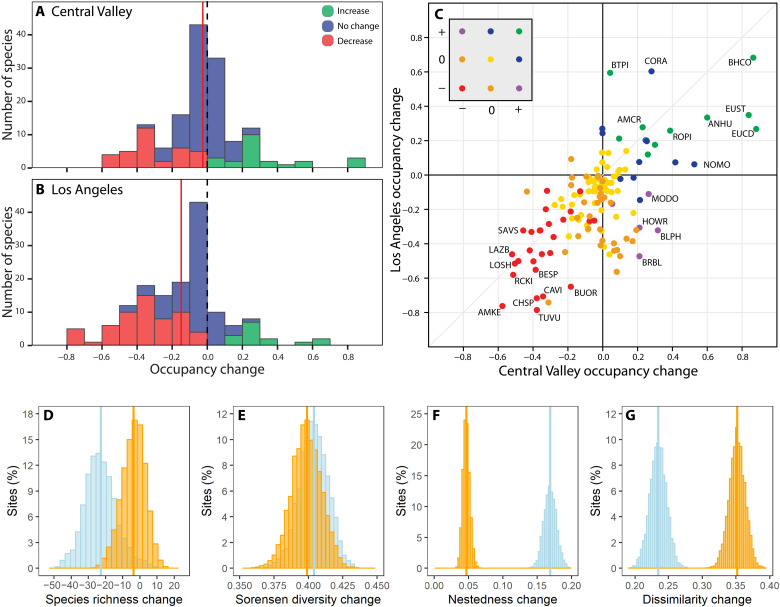
Changes in occupancy and diversity for 148 bird species in the Central Valley and Los Angeles over the past century. (**A** and **B**) Occupancy change in the Central Valley (A) and Los Angeles (B). Significant (95% CRIs for change not overlapping 0) increases are in green and decreases are in red, with blue for nonsignificant change. Red lines indicate the mean for a region. (**C**) Species-specific changes in occupancy in each region. Colors indicate significant or nonsignificant change in one or both regions (e.g., green represents significant increase in both regions). Four-letter species codes are in table S5. (**D** to **G**) Posterior distributions by region (orange, Central Valley; blue, Los Angeles) for changes per site in (D) species richness, (E) Sorensen’s overall dissimilarity index, (F) dissimilarity due to loss of species (nestedness), and (G) dissimilarity due to species replacement (Simpson’s index), with colored lines indicating distribution means. Overall dissimilarity (E) is the sum of (F) and (G).

Over the past century, the proportion of sites occupied (hereafter “occupancy”) declined more for birds inhabiting the urbanized Los Angeles region than the agricultural Central Valley (Fig. 2, A and B). In Los Angeles, mean (±SE) occupancy across the assemblage (historic, 0.43 ± 0.24; modern, 0.28 ± 0.02) declined by −0.15 [95% credible interval (CRI), −0.16 to −0.13], with 39.9% (*n* = 59) of the species significantly decreasing in occupancy, 10.1% (*n* = 15) significantly increasing, and 50.0% (*n* = 74) exhibiting no change. In contrast, the proportion of sites occupied in the Central Valley was relatively stable across the assemblage (historic, 0.32 ± 0.21; modern, 0.29 ± 0.02), and the mean change of occupancy per species was −0.03 (CRI, −0.04 to −0.01). In the Central Valley, 23.0% (*n* = 34) of the species decreased significantly in occupancy, 15.5% (*n* = 23) increased significantly, and 61.5% (*n* = 91) exhibited no significant change. Five species increased significantly in the Central Valley but decreased significantly in Los Angeles (Fig. 2C and table S2). In contrast, no species increased significantly in Los Angeles and decreased in the Central Valley. The top increasing species in both regions were predominantly non-native and native species that were tolerant of human habitat modification (table S2). Habitat preference was the primary trait associated with species-level changes in occupancy (table S3). Differences in the habitat used by species explained almost all of the variation in occupancy change [Akaike's information criterion corrected for small sample size (AICc) weight = 1.00] compared to body size, migratory behavior, diet, and tolerance of human habitat modification. The largest declines occurred in open-country species that use habitats with little tree cover in the Central Valley and in open-country and forest species in Los Angeles, while species preferring developed habitats and generalists increased similarly in both regions (fig. S2).

Urbanized Los Angeles experienced a larger loss of species over the past century than the agricultural Central Valley (Fig. 2, D to G). Average species richness in Los Angeles during the historic survey was 64.0 (CRI, 62.0 to 66.2) species per site and declined significantly in modern surveys by −22.8 (CRI, −25.1 to −20.5) species per site to 41.2 (CRI, 40.1 to 42.7) (Fig. 2D), a loss of over one-third of the species per site. In the Central Valley, however, species richness and assemblage composition at sites changed little over the past century. Historic species richness averaged 47.2 (CRI, 45.2 to 49.3) species per site and declined modestly by an average of −3.7 (CRI, −6.1 to −1.4) species per site to 43.5 (CRI, 42.3 to 45.0), an 8% decrease. Nevertheless, the overall change over the century in species composition (beta diversity) in the two regions (Fig. 2E) was similar (Sørensen’s dissimilarity index mean change: Central Valley, 0.40; CRI, 0.38 to 0.42; Los Angeles, 0.40; CRI, 0.38 to 0.42), although the underlying processes responsible for change differed greatly. Sites in Los Angeles had a significantly greater increase in nestedness (mean change, 0.17; CRI, 0.15 to 0.19) over time (Fig. 2F) due to a higher net loss of species compared to sites in the Central Valley (mean change, 0.05; CRI, 0.04 to 0.06), where replacement of one species by another or turnover (Fig. 2G) was the main cause of dissimilarity change (mean dissimilarity change: Central Valley, 0.35; CRI, 0.33 to 0.38; Los Angeles, 0.24; CRI, 0.21 to 0.26).

### Effects of climate and land-use change on avian turnover

**Fig. 3.**
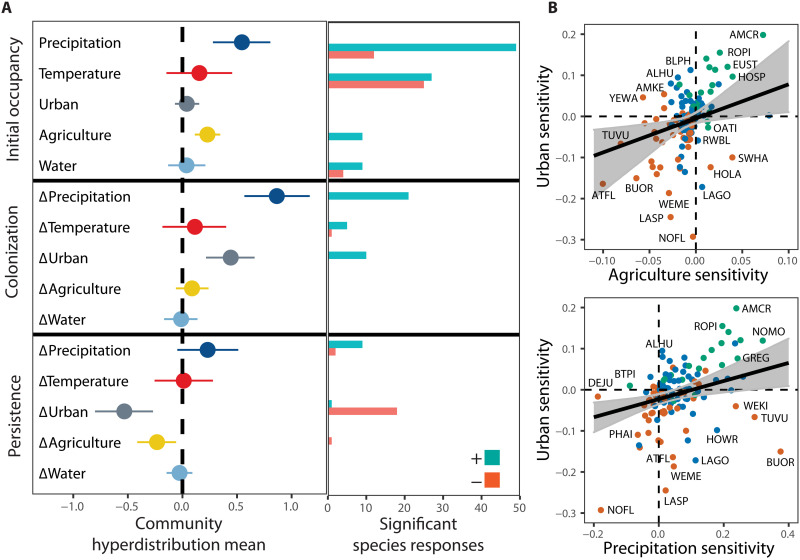
Effects of climate and land-use change on initial occupancy, colonization, persistence, and sensitivities of species. (**A**) Covariate coefficients of initial occupancy (early 1900s), and colonization and persistence (mean and 95% CRI) for community-level hyperparameters with the corresponding number of bird species with significant positive or negative effects. (**B**) Significant correlations among derivatives of occupancy change. Colors show significance of species-level occupancy change from historic to modern (red, decrease; green, increase; blue, not significant). Regressions with 95% CRIs incorporating species-level uncertainty are plotted. Four-letter species codes are in table S5.

The distribution of birds in Los Angeles and the Central Valley in the early 20th century was primarily associated with climate, but colonization and persistence at resurvey sites during the past century were determined by both land-use and climate change (Fig. 3A). Precipitation had the greatest effects on initial occupancy, and change in precipitation had the greatest impact on the colonization of unoccupied sites and the second largest effect on persistence of species at occupied sites. Species were at least four times more likely to have positive than negative associations with precipitation and precipitation change. While temperature affected the distribution of one-third of the species in the early 20th century, with roughly equal numbers of significant positive and negative effects, temperature change had less influence on turnover. Colonization was positively associated with temperature for five species and negatively associated for only one non-native species (table S2).

Urbanization had the strongest effects among land-use changes (Fig. 3A). Urbanization promoted colonization of 10 native and non-native species that benefitted from human habitation (table S2), but persistence was strongly negatively affected by urbanization at both the assemblage and species (*n* = 19) levels. Urbanization improved persistence for only the black phoebe (*Sayornis nigricans*). Agriculture had contrasting effects, being positively associated with initial occupancy, negatively associated with persistence at the assemblage level, and not significantly influencing colonization. Percent cover of water had little assemblage- or species-level influence on turnover but had mixed effects on species’ occupancy in the early 20th century.

### Sensitivity and impacts of climate and land-use change

Bird species exhibited both independent and correlated sensitivities to climate and land-use changes over the past century. Sensitivity provides a unified measure of both the direction (positive or negative) and magnitude (absolute value) of the change in occupancy in response to change of a climate or land-use covariate. Correlations among the derivatives of occupancy change (sensitivity) indicated species responded independently to precipitation and temperature change (fig. S3). Species that increased over the past century tended to have strong, positive sensitivities to precipitation change (fig. S4). The pattern was similar for temperature sensitivity, but the difference in sensitivity between species that increased and declined was smaller. In contrast, sensitivity of species to urbanization and agriculture was strongly positively related (Fig. 3B), and changes in occupancy were very sensitive to changes in urban cover (fig. S4). Birds were least responsive to changes in water cover; it had a mean sensitivity value near zero and the smallest range. Other correlations among sensitivity measures were not significant (fig. S3).

Species’ sensitivities to climate and land-use change were unrelated with one important exception (Fig. 3B and fig. S3); there was a positive correlation between precipitation sensitivity and urban sensitivity. Species exhibiting occupancies that increased with urbanization (positive urban sensitivity) were more likely to increase at sites that became wetter (positive precipitation sensitivity), while species that decreased with urbanization (negative urban sensitivity) were more likely to decrease at sites that became wetter (negative precipitation sensitivity). Occupancy of most species responded positively to increased precipitation, but responses to urbanization were more evenly split between positive and negative. Given that changes in precipitation and urban cover are the two most impactful threats affecting species’ persistence and colonization (Fig. 3A), this result suggests that climate and land-use change could combine to produce occupancy change, with strong impacts in regions experiencing urbanization and drying.

**Fig. 4.**
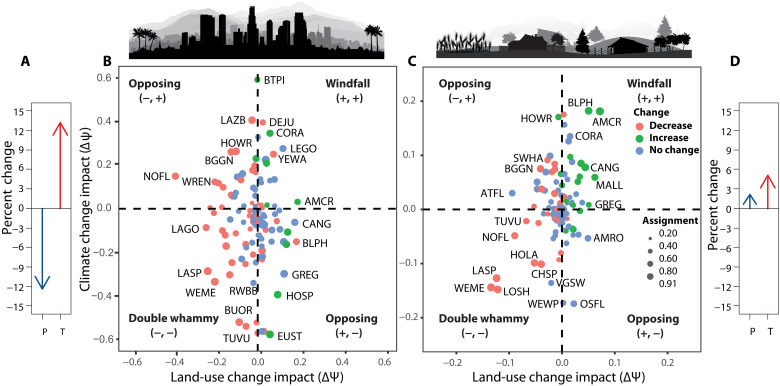
Independent impacts of land-use and climate change on bird species occupancy change over the past century. (**A** and **D**) Vectors of the mean percent change in average annual precipitation (P) and temperature (T) for Los Angeles (A) and the Central Valley (D). (**B** and **C**) Mean impact on bird species in Los Angeles (B) and the Central Valley (C). Quadrats associated with concordant (windfall and double-whammy) and opposing effects of climate and land-use change are labeled. Colors indicate significance of occupancy change over the past century, and symbols are scaled by the probability of assignment to quadrats. Four-letter species codes are in table S5. Nine non-native or invasive species not present during the early 20th century surveys were excluded.

Bird species exhibited both concordant and opposing responses to climate and land-use change over the past century, as quantified by counterfactual impacts estimated for climate change in the absence of land-use change and vice versa (Fig. 4). Species with concordant responses to land-use and climate change were slightly more common than those experiencing opposing effects (Los Angeles, 53% versus 47%; Central Valley, 58% versus 42%). In Los Angeles, where urbanization was severe and the climate strongly warmed and dried, 39% of species experienced the double whammy of negative impacts from both land-use and climate change on occupancy, while only 14% experienced positive windfall impacts from both threats. In the Central Valley, species experiencing windfalls from both threats were more common (24%) and nearly offset those suffering double-whammies (34%). Climate and land-use change impacts across species in a region were not significantly correlated [Los Angeles: correlation coefficient (*r*) = −0.055; CRI, −0.189 to 0.088; Central Valley: *r* = 0.025; CRI, −0.183 to 0.238].

## DISCUSSION

Despite similar species composition, geographic proximity, and long histories of habitat modification, birds of the Central Valley and Los Angeles experienced very different patterns of change over the past century. We found evidence for both decline and stability of occupancy and species richness for birds (Fig. 2) caused by differences in how the combination of climate and land-use change affected persistence and colonization (Fig. 3). Sites in Los Angeles historically supported greater avian species richness than those in the Central Valley but, over the past century, have experienced larger declines in species occupancy and diversity. Today, avian diversity in Los Angeles is similar to the Central Valley, although our results are confined to breeding bird assemblages and do not address the impact of wetland loss in the Central Valley on migratory and some resident water birds that were not sampled adequately. Our findings confirm that climate and land-use change are not universally accompanied by biodiversity loss but, instead, may result in decline (*34*, *35*) or stability (*36*, *37*) depending on the geographic context and scale (*38*, *39*), the level of exposure to each threat, and species’ sensitivities (*12*, *14*).

Over the past century, the distribution of birds of Los Angeles and the Central Valley shifted from being shaped predominately by climate to being shaped by the combined forces of land use and climate. Historically, strong effects of temperature and precipitation on occupancy (Fig. 3A, initial occupancy) likely reflected the adaptations of species to the semiarid climate shared by these regions and the minimal effects of land use, as evidenced by limited urban and agricultural cover (Fig. 1). In contrast, urbanization was a major driver of turnover in the early 21st century, strongly positively affecting colonization of some species and negatively affecting persistence of many more species (Fig. 3A), while agriculture had a weaker effect, primarily reducing persistence. The 36% decline in species richness per site in Los Angeles (Fig. 2) is similar in magnitude to the avian community collapse in the nearby Mojave Desert over the same time period due to climate change (*14*, *40*). Despite substantial warming at or nearly double the average increase in global mean surface temperature (1° ± 0.2°C) (*41*) in the Central Valley and Los Angeles, respectively, temperature change had little influence on turnover compared to change in precipitation (Fig. 3A). It was nearly as influential as urbanization in shifting the distribution of birds in these regions (Fig. 3A).

A key finding from this study is that bird species exhibited both concordant and opposing responses to climate and land-use change over the past century. As a result, climate change both amplified and moderated the effects of land-use change on the same species pool, depending on the combination of change that each region experienced. Climate change in the agricultural Central Valley brought wetter conditions that promoted colonization of unoccupied sites and enhanced persistence at occupied sites, which acted to partially moderate the negative effects of intensive land-use change on avian diversity (Figs. 2 to 4). In contrast, climate change amplified avian declines in urbanizing Los Angeles, where drier conditions reduced site-level persistence and colonization. This created a double jeopardy for birds because occupancy change for many species responded negatively to increased urbanization and drying, and sensitivity to urbanization and precipitation was positively related (Fig. 3B). Almost three times as many bird species in Los Angeles experienced a double whammy of negative impacts on occupancy from both land-use and climate change as experienced positive windfall impacts from both threats, compared to the Central Valley where windfalls were more common and nearly offset fewer species suffering double-whammies (Fig. 4).

Quantifying the concordant and opposing responses of species to threats (Fig. 4), as well as the resulting effect on assemblages, expands the concept of cotolerance (*7*) by demonstrating that a broader set of responses occurs (*8*). In particular, opposing responses to climate and land-use change were about as common as concordant responses (Fig. 4). Thus, it was expected that climate and land-use impacts across an assemblage of species in a region were not significantly correlated. We expect that opposing impacts of stressors may be common for some combinations of threats and exposure, leading to a tension in species’ responses depending on the magnitude of exposure to each threat and the sensitivity of species. For example, climate change over the past century in a montane region of northern California resulted in opposing impacts, causing a “push” upward from warming and “pull” downward from wetting on the elevation limits of birds as they tracked their climatic niches (*42*). Moreover, in Los Angeles and the Central Valley, concordant responses led to both occupancy increases and decreases over the past century, depending on whether correlated responses to climate and land-use change resulted in windfall or double-whammy impacts. We have not considered the order of exposure to threats, which can affect responses (*43*). In our study land-use change primarily occurred before the onset of climate change in the 1970s and its acceleration in the 2000s, which may be the likely order of occurrence in many long-settled temperate regions. However, the order may be reversed in tropical regions undergoing strong land conversion (*23*).

In conclusion, the impacts of contemporary climate and land-use change (Figs. 2 and 4) will depend on the particular combination of regional exposure to each threat that occurs (Fig. 1) and the sensitivity of species (Figs. 3 and 4). The combinations of climate and land-use change that occurred in Los Angeles and the Central Valley resulted in concordant and opposing responses of species that amplified and moderated impacts, respectively, in these regions. These processes may promote unpredictable feedbacks and synergies (*11*, *44*) that complicate projections of species distributions and predictions of extinction risk for biodiversity (*45*).

## MATERIALS AND METHODS

### Study area

We studied changes in bird diversity in two of the ecoregions most transformed by climate and land-use change in California (*31*, *46*): the Central Valley and Los Angeles (fig. S1). Together, they extend approximately 1000 km from north to south and span an elevation gradient from sea level to 2500 m.

The Central Valley study region included the low, flat Great Valley ecoregion and adjacent portions of the foothills in the Central Valley Coast Ranges ecoregion on the west and Sierra Nevada Foothills ecoregion on the east (*47*). Survey sites ranged from the valley floor into the surrounding foothills. The natural vegetation is a mosaic of seasonal wetlands and riparian belts surrounded by grassland, oak woodland along the foothills, and saltbrush scrub in the southern valley (*48*). Since the early 20th century, the Central Valley has become one of the most productive agricultural regions in the world, with approximately 70% of its area under cultivation (*46*). Moreover, urban areas in the Central Valley have also grown at one of the fastest rates in California (*49*). Compared to other ecoregions in California, the Central Valley has the lowest percent (16%) of untransformed lands remaining (*46*) and experienced a greater than average increase in mean annual temperature over the 20th century (*31*).

The Los Angeles study region was composed of portions of the Greater Los Angeles metropolitan area and the surrounding foothills within Los Angeles County as well as the adjacent Angeles National Forest and the San Bernardino National Forest, which lies at the extreme southwest corner of San Bernardino County. The Los Angeles study area includes portions of the Southern California Coast and the Southern California Mountains and Valleys ecoregions (*47*). It is separated from the Central Valley by the Transverse Ranges to the north and bordered by the Pacific Ocean to the west and by the Peninsular Ranges to the east. The Southern California Coastal ecoregion is a hot spot for rare species in the United States (*50*), and Los Angeles County hosts the greatest number of bird species of any U.S. county (*51*). The natural vegetation is predominantly chaparral and riparian. Los Angeles differs from the Central Valley in the former’s relative scarcity of grassland, as well as greater coverage of coniferous woodland at higher elevation. Little agricultural development has occurred in Los Angeles over the past century, while urbanization dominates the valley floor. The Greater Los Angeles metropolitan area has grown to be the second-most populous in the United States (*49*). Compared to other ecoregions in California, the Los Angeles study region experienced the greatest increase in mean annual average temperature except for the Sonoran Desert, which had little land-use change (*46*), and experienced the largest reduction in annual precipitation during the 20th century (*31*).

### Site selection and avian survey methods

To obtain data on historic localities and bird species occurrences, we reviewed original field notebooks written by Joseph Grinnell and several of his colleagues, which are curated by the Museum of Vertebrate Zoology at University of California, Berkeley. These field notebooks provide detailed descriptions and maps of survey routes, as well as systematic lists of bird species observed each day. We identified 71 sites (Central Valley, 43; Los Angeles, 28; fig. S1) with historic surveys of bird diversity that sampled representative habitats and climates throughout the range of each study region. Central Valley sites extended the length of the valley (~640 km) and ranged from 2 to 1817 m above sea level (masl) (*48*), while Los Angeles sites ranged from near the downtown region to the upper elevations of the San Bernardino National Forest (13 to 2333 masl) covering ~150 km.

We collected historic and modern bird survey data following standardized protocols for the Grinnell Resurvey Project that have been described in detail elsewhere (*40*, *42*, *48*). Bird surveys by Grinnell and colleagues were carefully documented in the form of field notebooks, museum specimens, photographs of sampling sites, and annotated topographic maps deposited at the Museum of Vertebrate Zoology at University of California, Berkeley, permitting identification of historic survey locations with a high degree of accuracy (*52*). Historic surveys occurred from late March through July between 1895 and 1908 in Los Angeles and from 1912 to 1923 in the Central Valley. They were occasionally in the form of standardized abundance surveys that were precursors to modern line transects, more often as lists of species encountered each day that provide detection/nondetection data, and rarely as a daily list that identified only species that had not been detected previously. All three kinds of data could be used in our occupancy model. Each site had an average of 3.15 consecutive days of historic surveys (range, 1 to 11 days). We conducted modern resurveys during the breeding seasons (April to July) of 2015–2017, matching the following characteristics of the historic surveys: geographical location and extent of survey sites, elevational range covered, habitats surveyed within sites, and timing of the survey during the breeding season. We used standardized variable-distance point counts along a 2.25-km transect, with 10 points placed 250 m apart, corresponding as closely as possible to the area and habitats noted by the historic surveyors and indicated by specimen collecting locations. Surveys began at dawn and lasted 2 to 3 hours. At each point along the transect, we recorded all birds seen or heard during a 7-min period. Each site was surveyed daily over three consecutive days.

Bird counts from modern surveys were collated for each day across all 10 points surveyed along a transect and reduced to detection/nondetection data per day per site for occupancy modeling. Surveys that spanned multiple years were appended as subsequent visits into a single detection history. Occupancy in our study should be interpreted as occupancy at a site during the span of years sampled and any changes in occupancy within those years get absorbed in detection probability. Population size fluctuations across years are much greater than site-level changes in occupancy, especially at the scale that we have defined sites. The duration of the time over which sites were sampled in the Central Valley and Los Angeles is nearly the same (13 years versus 11 years) and is small compared to the elapsed time between survey eras (about a century).

A total of 148 bird species were included in our analysis that were known to breed in the Central Valley or Los Angeles (*53*). We included species detected flying by only if they remained within the observable area (e.g., circling or flying low over the vegetation), including six species of diurnal raptors. Raptors had a high mean detection probability (*p*) by our modern point count transects (*p* = 0.53 to 0.69 per visit), yielding a high site-level detection probability (p*) from three survey visits (p* ranged from 0.89 to 0.97), with the exception of the golden eagle that was lower (*p* = 0.24, p* = 0.56). See data S1 for detection probabilities and scientific names of species included in the study. Following (*48*), we excluded nocturnal birds (owls and nightjars) and obligate wetland birds because they were not sampled adequately by our survey protocol or by historic surveyors. We excluded two species of shorebirds (American avocet *Recurvirostra americana* and black-necked stilt *Himantopus mexicanus*), one wading bird (white-faced ibis *Plegadis chihi*), and eight species of waterfowl (wood duck *Aix sponsa*, northern pintail *Anas acuta*, gadwall *Mareca strepera*, cinnamon teal *Spatula cyanoptera*, ruddy duck *Oxyura jamaicensis*, western grebe *Aechmophorus occidentalis*, Clark’s grebe *Aechmophorus clarkii*, and pied-billed grebe *Podilymbus podiceps*). The nine species of water birds that we retained had strong connections with upland or riparian habitats that were well sampled in this study. They included belted kingfisher, black-crowned night heron, Canada goose, great blue heron, great egret, green heron, killdeer, mallard, and snowy egret. Detection probability per visit (*p*) for these species ranged from 0.49 (black-crowned night heron) to 0.83 (killdeer), with *p* averaging 0.66 and p* averaging 0.95 across the nine included water birds. We also excluded several non-native species that were detected at only a single site during modern surveys due to limited population establishment, including the rose-ringed parakeet (*Psittacula krameri*) in the Central Valley and the northern cardinal (*Cardinalis cardinalis*), Egyptian goose (*Alopochen aegyptiaca*), blue-fronted parrot (*Amazona aestiva*), and scaly-breasted munia (*Lonchura punctulata*) in Los Angeles.

### Climate and land-use change covariates

To characterize climate (long-term average weather conditions often presented as 30-year climate normals), data were obtained from 800-m resolution interpolated maps produced by the PRISM Climate Group (*54*) and averaged over 30-year periods corresponding to the historic (1900–1929) and modern (1988–2017) surveys. Annual minimum, maximum, and average temperatures were extracted for each bird survey point at a site during the 30-year historic time period, averaged across all points to acquire annual means for each site, and then averaged to yield the historic climate values for a site over the 30-year period. The same process was followed for the 30-year modern time period. Annual minimum, maximum, and average temperatures at our survey sites were highly correlated, so we chose to use annual average temperature in the interest of parsimony. We used total annual values for precipitation. Climate change was represented as the difference between modern and historic climate covariates.

To quantify land-use change (modern minus historic), we followed the methods of MacLean *et al.* (*48*) and created maps of historic land use within 1 km of our bird survey transects by hand-digitizing historic maps using ArcMap for comparison with modern land-use data obtained from the National Land Cover Database (NLCD) (*55*). Water and urban area were hand-digitized from historic U.S. Geological Survey topographic maps (ca. 1906–1932). Water bodies were directly outlined as polygons. Urban area was mapped as buildings (area of the building icon on the topographic map plus a buffer of 50 m) and roads (digitized as line features from the topographic map and given a width of 30 m). Historic agriculture was delineated using a series of three maps of irrigated lands in California in 1920 (*56*). Areas that did not fall into one of these three land-use categories were assumed to be natural land cover, which likely included a mix of untransformed and lightly transformed lands. We converted our digitized historic land use from vector format to raster format at 30-m resolution per pixel, corresponding to NLCD dataset. To make historic and modern land-use types comparable, modern urban combined four “developed” NLCD categories (high, medium, low, and open space), modern agriculture consisted of NLCD categories of “cultivated crops” and “pasture/hay,” and water consisted of open water for both eras. We then calculated changes in percent of urban land, agricultural land, surface water, and natural land at buffers of 100 m, 200 m, 500 m, and 1 km around the survey transect composed of the points used to resurvey birds.

Our choice of focal land-use categories represents the dominant land-conversion processes that have occurred in the Central Valley and Los Angeles over the past century (*55*). Change among natural land-cover types (i.e., grassland, wetland, scrub, riparian, and woodland) could not be directly quantified because of lack of historic data at relevant spatial and temporal resolutions. However, coarser-resolution mapping projects (*19*) and qualitative descriptions in the historic field notes suggest that there have been limited transitions among natural land-cover types since the early 1900s. Therefore, we believe that our focal land-use categories adequately capture the processes of change most relevant to avian occupancy.

### Dynamic multispecies occupancy model

We used a dynamic multispecies occupancy model (MSOM) to estimate the probabilities of occupancy (ψ), local colonization (γ), and local persistence (ϕ) between the historic and modern survey periods (*57*). MSOMs are commonly used to quantify metacommunity dynamics (*40*, *57*) and metrics of diversity (*58*–*60*) because they account for imperfect detection of species while producing robust estimators of occupancy (*61*). The MSOM estimates species-specific values for each parameter assuming parameters come from shared, community-level distributions, each with hyperparameters (*59*), which is equivalent to including a species-level random effect. Our notation below is adapted from Iknayan and Beissinger (*40*) and Riddell *et al.* (*14*) and used the code by Iknayan and Beissinger (*40*), which is presented in detail in their Supplementary Materials and is included with our data repository for this article.

Bird survey data *y_ijkt_* (1 if detected and 0 if not detected) for the *i*th species at the *j*th site on the *k*th visit in the *t*th time period were assumed to result from imperfect observation of the true incidence *z_ijt_* (1 if present and 0 if absent). The probability of the survey data given true incidence and detection probability (*p_ijkt_*) was

$$y_{ijkt}|z_{ijt},P_{ijkt}∼\mathrm{Bernoulli}(z_{ijt}P_{ijkt})$$ (1)

Initial incidence *z*_*ij*1_ was 1 with probability ψ_*ik*1_ of initial (historic) occupancy probability

$$z_{ij1}|\psi_{ij1}∼\mathrm{Bernoulli}(\psi_{ij1})$$ (2)

Probability of local persistence and colonization from the historic to the modern survey period were modeled as a first-order Markovian process, so that species incidence in the modern survey period (*z*_*ij*2_) was dependent on that species’ incidence in the historic period (*z*_*ij*1_). Incidence during the modern time period at a site was modeled as a function of species’ probability of local persistence (ϕ*_ij_*) and local colonization (γ*_ij_*) as follows

$$z_{ij2}|z_{ij1},\phi_{ij},\gamma_{ij}∼\mathrm{Bernoulli}[\phi_{ij}z_{ij1}+\gamma_{ij}(1-z_{ij1})]$$ (3)

Each of the four probabilities (detection, initial occupancy, persistence, and colonization) was modeled as a linear combination of site and/or survey period covariates using a logit-link transformation

$$\mathrm{logit}(p_{ijkt})=\alpha_{0i}+W_{jkt}{\text{α}}_{i}$$ (4)

$$\mathrm{logit}(\psi_{ij1})=\beta_{0i}+X_{j}{\text{β}}_{i}$$ (5)

$$\mathrm{logit}(\phi_{ij})=\delta_{0i}+Y_{j}{\text{δ}}_{i}$$ (6)

$$\mathrm{logit}(\gamma_{ij})=\epsilon_{0i}+Z_{j}{\text{ϵ}}_{i}$$ (7)

where naught terms represent the species-specific intercept for each probability, bold Greek variables are vectors of species-specific coefficients, and bold Latin variables are matrices of the associated covariates, with indices for one row. For *p*, we allowed detection to vary during the breeding season as a function of Julian day (= 1 on 1 January) and its quadratic, because singing by birds for territory and mate acquisition and, hence detectability, often follows this pattern (*40*, *62*). To account for expected differences in detection between historic and modern surveyors due to differences in survey methods and advances in ornithological knowledge and technology (*63*), we also modeled detection probability as a function of survey era (categorical). Initial occupancy (ψ_1_) was modeled as a linear function of average annual temperature, annual precipitation, percent cover of water, percent cover of urbanization, and percent cover of agriculture. Probabilities of persistence and colonization were both modeled as a function of the change (modern minus historic) in temperature, precipitation, and land use (water, urban, and agriculture). All continuous covariates were centered at 0 and normalized to an SD of 1 to produce *z* scores for scaling before analysis.

We used uninformative priors for the hyperdistributions of the intercept terms and means of the coefficients and weakly informative priors for the variances of the hyperdistributions as a type of regularization known as shrinkage to regularize parameter estimates for species that were sparsely observed (*64*). To accomplish these goals, we followed recommendations (*65*, *66*) and, as with our previous implementation (*40*), set the hyperdistribution for community-level priors as Normal(0, 2.25) for means and as half-Cauchy for SDs using the Student’s *t* distribution with df set to one *T*(0, 2.25, 1) and censored the distribution above zero *T*(0, ).

Data were preprocessed in R, and Bayesian parameter estimation was implemented using Markov chain Monte Carlo (MCMC) in JAGS via the R package “jagsUI.” Models were fully adapted (*n* = 200). We ran 12 parallel chains of length 65,000 discarding the first 40,000 as burn-in and used a thinning rate of 50. This resulted in a posterior distribution consisting of 6000 samples for each parameter. Convergence was assessed by visual inspection of trace plots and by using the Gelman-Rubin convergence diagnostic (*64*). All diagnostic values were ≤1.1, which satisfies the criteria for convergence. Outputs for each species and region are summarized in data S1.

To determine the most informative spatial scale for land-use covariates, separate models were run at each scale (100 m, 200 m, 500 m, and 1 km). The 1-km scale was selected on the basis of Watanabe-Akaike information criterion (WAIC) (*64*), as it outperformed all other scales by 16 to 114 WAIC points (table S4).

MSOMs assume that (i) sites are “closed” to emigration and immigration of species between surveys within a sampling period, (ii) species are accurately identified (i.e., no false positive detections), (iii) the probability of detecting a species is independent among sites, and (iv) random effects for species are drawn from the same distribution (*61*). Violating these assumptions would affect our inferences only if they affected historic and modern survey data differentially, which is unlikely for our study. To reduce the chance of violating the closure assumption, historic and modern survey data were collected at similar temporal and spatial scales. Closure violations due to temporary emigration are more likely to occur with small sampling units (e.g., individual point counts) that may intersect only fractions of animal territories (*67*) than the large sampling units that characterize this study (10 point counts aggregated across a 2.25-km transect). Modern bird surveys were completed within the same year, usually over three consecutive days. Historic surveys were mostly conducted within a week in the same year (83%) or in consecutive years (14%), although two sites were sampled at longer intervals. Any violation of closure at the site level from surveys over this span of elapsed time seems likely to be small. Because sites were not close to one another relative to the home ranges of birds, the probability of detecting a species at sites should be independent. See appendices in (*14*) for further discussion on applying the MSOM framework to Grinnell Resurvey data and see (*42*) for evidence that bias from violating the closure assumption is unlikely to affect conclusions about occupancy change based on statistical simulations with Grinnell Resurvey data.

### Species traits

We tested the strength of traits as predictors of mean change in species’ occupancy between the historic and modern survey periods. We focused on five frequently tested and supported traits relevant to changing species’ distributions: habitat use, diet, migratory behavior, log-transformed body size, and tolerance for human habitat modifications (*68*, *69*). The complete dataset of species’ traits is presented in data S2. Trait data were obtained from *The Birds of North America Online* (*70*), the online Encyclopedia of Life (*71*), and Elton Traits (*72*).

We modeled change in species-specific occupancy as a function of each individual trait using linear mixed effects models, with species as a random effect. To account for model uncertainty, we weighted each measure of mean occupancy change by the inverse variance of its posterior distribution. Models with combinations of two or more traits never performed better than single-trait models, so we excluded these from our final model set. We compared the full model set using AICc (table S3).

### Species diversity

We calculated changes in species richness and beta diversity between the historic and modern survey periods for the sites in each region. All metrics were calculated using latent incidence *z_ijt_* (for the *i*th species at the *j*th site in a region in the *t*th time period) as estimated from the MSOM and repeated for all 6000 samples of the posterior distribution to propagate model uncertainty.

Species richness *N*_*j*,*t*_ was calculated simply as the sum of species per site

$$N_{j,t}=\sum_{i=1}^{148}z_{ijt}$$ (8)

We quantified the degree that avian species composition changed at sites over time and whether changes were the result of species loss (i.e., nestedness) and turnover, which is the replacement of species by other species. Following (*73*) and using the beta.temp command of the betapart package in R (*74*), we calculated three partitions of beta diversity that reflect these processes for each posterior sample of the true incidence matrix, *z_ijt_*, for each site: (i) total change in beta diversity (i.e., the sum of nestedness and turnover) measured by Sørensen’s dissimilarity index

$$\beta_{\mathrm{sor}}=\frac{b+c}{2a+b+c}=\beta_{\mathrm{nested}}+\beta_{\mathrm{sim}}$$ (9)

(ii) change in beta diversity caused by loss of species, measured by the nested-resultant dissimilarity index

$$\beta_{\mathrm{nested}}=\frac{\max(b,c)-\min(b,c)}{2a+\min(b,c)+\max(b,c)}\times \frac{a}{a+\min(b,c)}$$ (10)

and (iii) change in beta diversity due to turnover measured by Simpson’s dissimilarity index

$$\beta_{\mathrm{sim}}=\frac{\min(b,c)}{a+\min(b,c)}$$ (11)

where *a* is the number of bird species detected at a site in both historic and modern surveys, *b* is the number of species detected in historic but not modern surveys, and *c* is the number of species detected in modern but not historic surveys.

### Sensitivity and counterfactuals to quantify impacts of climate and land-use change

The MSOM described above was structured to model modern occupancy (ψ_m_) by estimating the effects of climate and land-use covariates on historic (initial) occupancy (ψ_h_), colonization, and persistence (Eq. 3) using the Markovian form commonly found in dynamic occupancy models

$$\psi_{m}=\psi_{h}\phi +(1-\psi_{h})\gamma$$ (12)

Here, eras “h” and “m” correspond to *t* = 1 and *t* = 2 above, respectively. Thus, to understand how change in a covariate affected occupancy change, we created a metric of covariate effects on both persistence (at sites that were occupied historically) and colonization (at sites that were unoccupied historically).

To do this, we quantified the sensitivity of each species to climate and land-use change as the derivative of the change in occupancy (Δψ = ψ_m_ − ψ_h_) for a species with respect to the change in a climate or land-use covariate (e.g., change in precipitation Δ*P* = *P*_m_ − *P*_h_). It can be expressed as the combination of terms from persistence and colonization

$$d\text{Δ}\psi /d\text{Δ}P=\psi_{h}[\phi (1-\phi )\alpha_{1}]+(1-\psi_{h})[\gamma (1-\gamma )\beta_{1}]$$ (13)

where α_1_ and β_1_ are the covariate’s slopes for persistence and colonization, respectively. The change in occupancy with respect to a change in a climate or land-use covariate can be positive or negative. A positive number indicates that a species increased in occupancy with a positive change in the covariate, while a negative number indicates that it decreased in occupancy with a positive change in the covariate. The absolute value of the derivative indicates the magnitude of sensitivity. See Supplementary Text for the derivation of Eq. 13 and table S5 for derivatives of occupancy change for each species and covariate, quantified from the full posterior of the MSOM using Eqs. 6 and 7.

We tested for evidence of phylogenetic signal for sensitivity measures by downloading 1000 trees with the Ericson backbone from birdtree.org. Of the five tests (Abouheif’s *C*_mean_, Moran’s *I*, Blomberg’s *K* and K*, and Pagel’s λ) implemented on an average tree and a 50% majority rule consensus tree with the R packages “caper” and “phylosignal” (*75*), only water sensitivity showed evidence of phylogenetic dependence (table S6), so we report results uncorrected for phylogeny. When models were rerun using pgls on the average tree, results remained the same.

We used counterfactuals to dissect the independent impacts of climate and land-use change on each species in each region. Occupancy was modeled for three scenarios for each region: (i) region-specific climate change (ψ_CC_) in the absence of land-use change; (ii) regional land-use change (ψ_LU_) in the absence of climate change; and (iii) the absence of both land-use and climate change, which served as a control (ψ_CT_). ψ_CT_ measures occupancy change due to unmodeled factors (e.g., disease and introduced predators) in each region.

Impacts on occupancy (Δψ) were then calculated for each threat in each region by subtracting the control: Δψ_CC_ = ψ_CC_ − ψ_CT_ and Δψ_LU_ = ψ_LU_ − ψ_CT_. Use of a control in this manner avoids problems from not accounting for the effects of other causes of occupancy change that are baked into the intercept terms in Eqs. 6, 7, 14, and 15 (*76*).

To calculate ψ_CC_, ψ_LU_, and ψ_CT_, we combined Eqs. 12, 6, and 7. Specifically, species-specific Eqs. 6 and 7 are written explicitly as

$$\phi_{i}={\mathrm{logit}}^{-1}(\alpha_{0i}+\alpha_{1i}\text{Δ}P+\alpha_{2i}\text{Δ}T+\alpha_{3i}\text{Δ}A+\alpha_{4i}\text{Δ}U+\alpha_{5i}\text{Δ}W)$$ (14)

$$\gamma_{i}={\mathrm{logit}}^{-1}(\beta_{0i}+\beta_{1i}\text{Δ}P+\beta_{2i}\text{Δ}T+\beta_{3i}\text{Δ}A+\beta_{4i}\text{Δ}U+\beta_{5i}\text{Δ}W)$$ (15)

where α_0*i*_ and β_0*i*_ represent the mean species-specific intercept for each probability and the delta terms and their associated slopes relate to the change over the past century in climate (*P* = precipitation and *T* = temperature) and land-use (*A* = agriculture, *U* = urban, and *W* = water).

Calculations were carried out with the full posterior of the MSOM using the parameter estimates for the *α* and β terms in Eqs. 14 and 15 for each species and the regional estimates of ψ_h_ for each species based on the incidences in Eq. 3. We set the *z* scores of the Δ values of the scaled covariates modeled for climate or land-use change to regional averages of change, while setting the *z* scores of the “no-change” covariates to regional conditions in the historic era (table S7). Because continuous covariates were centered at 0 and normalized to one SD for the pooled regions, we determined the *z* scores associated with the mean change and no change conditions for land-use and climate covariates for each region (table S7). For example, to model ψ_LU_ for Los Angeles, we calculated γ_LU_ and ϕ_LU_ with Eqs. 14 and 15 by setting the *z* scores of Δ*P* and Δ*T* to no change values, namely, those of the historic conditions (−2.471 and 0.455, respectively), while assigning *z* scores to the values of regional change (modern and historic conditions) that occurred for Δ*A*, Δ*U*, and Δ*W* (−0.548, 0.531, and 0.029, respectively). The full set of equations is provided in Supplementary Text.

Mean impact for each species for each threat was assigned to one of the four quadrants (Fig. 4) based on windfall (+, +), double whammy (−, −), or opposing (−, + or +, −) effects of land-use and climate change. Uncertainty in quadrat assignment was quantified by the proportion of the posterior samples that fell into the same quadrat as the mean.

Impacts were tested for phylogenetic signal as described above. There was little evidence of dependency (table S6), so standard statistical models were used.
