# Supplementary material for: Concordant and opposing effects of climate and land-use change on avian assemblages in California’s most transformed landscapes
Source: Sci Adv. 2023 Feb 22;9(8):eabn0250. doi: 10.1126/sciadv.abn0250 (PMC9946348; doi:10.1126/sciadv.abn0250)
Supplement: Supplementary file 1 — Supplementary Text Figs. S1 to S4 Tables S1 to S7 Legends for data S1 and S2 [file sciadv.abn0250_sm.pdf]

Science Advances
AAAS

Supplementary Materials for

Concordant and opposing effects of climate and land-use change on avian assemblages in California's most transformed landscapes

Steven R. Beissinger *et al.*

Corresponding author: Steven R. Beissinger, beis@berkeley.edu

Sci. Adv. **9**, eabn0250 (2023)
DOI: 10.1126/sciadv.abn0250

The PDF file includes:

Supplementary Text
Figs. S1 to S4
Tables S1 to S7
Legends for data S1 and S2

Other Supplementary Material for this manuscript includes the following:

Data S1 and S2

Supplementary Text

Derivation for the rate of change of occupancy with respect to a covariate

In this section we derive the equation [13] in the main text for the rate of change of modern occupancy with respect to a covariate of climate or land-use change, denoted generically as x, in the standard dynamic multi-season occupancy model.

The probability that a site is occupied by a species in the modern era multi-season occupancy model is

$$\psi_m(x) = \psi_h \phi(x) + (1 - \psi_h)\gamma(x) \quad [1]$$

where ψ_h is the probability of occupancy in the historic period, which does not depend on climate or land-use change, $\phi(x)$ is the probability of persistence, and $\gamma(x)$ is the probability of colonization.

Logistic relationships are used for persistence and occupancy probabilities, so we have

$$\phi(x) = \phi\big(\eta(x)\big) = \frac{e^{\eta(x)}}{1+e^{\eta(x)}} \quad [2]$$

where $\eta(x) = \alpha + \beta x$. By the chain rule (and algebra to rearrange factors in convenient forms), the derivative of $\phi(x)$ with respect to x is

$$\frac{d\phi(x)}{dx} = \phi(x)\big(1 - \phi(x)\big)\beta \quad [3]$$

Using a similar relationship for $\gamma(x)$ and combining the terms for $\psi_m(x)$ gives

$$\frac{d\psi_m(x)}{dx} = \psi_h \phi(x)\big(1 - \phi(x)\big)\beta_\phi + (1 - \psi_h)\gamma(x)\big(1 - \gamma(x)\big)\beta_\gamma \quad [4]$$

where β_ϕ is the slope of $\eta(x)$ for persistence probability and β_γ is the slope of $\eta(x)$ for colonization probability.

Counterfactual Equations for independent impacts of climate and land-use change

Occupancy was modeled for three counterfactual scenarios: (*i*) region-specific climate change (ψ_{CC}) in the absence of land-use change, (*ii*) regional land-use change (ψ_{LU}) in the absence of climate change; and (*iii*) the absence of both land-use and climate change, which served as a control (ψ_{CT}) for unmodeled factors (e.g., disease, introduced predators, etc.) in each region. The control was not region-specific because climate and land-use change covariates were centered and scaled across both regions in the Multispecies Occupancy Model (MSOM).

Equations [14,15] in the main text were implemented for each scenario, setting the z-scores of the delta values of the scaled covariates that were modeled for climate or land-use change to regional averages and the z-scores of the covariates held constant to match the conditions in the early 20th century. These values are given in Table S7. Note that the delta terms (levels of covariate change) are region-specific but the slopes for each covariate's effects on colonization

and persistence are not region-specific because they are estimated in the MSOM using the data pooled across both regions.

We write out the full equations for the general form of the dynamic multi-season occupancy model with each land-use and climate change covariate below:

$$\psi_m = \psi_h \phi + (1 - \psi_h)\gamma \quad [5]$$

which is implemented in the MSOM as

$$z_{ij2}|z_{ij1}, \phi_{ij}, \gamma_{ij} \sim Bernoulli\left(\phi_{ij1} z_{ij} + \gamma_{ij}\left(1 - z_{ij1}\right)\right). \quad [6]$$

Logit links were used for relating colonization and persistence probabilities to changes in land-use and climate variables. These equations with the full set of climate and land-use change covariates are:

$$\phi_i = \text{logit}^{-1}\,(\alpha_{0i} + \alpha_{1i}\Delta P + \alpha_{2i}\Delta T + \alpha_{3i}\Delta A + \alpha_{4i}\Delta U + \alpha_{5i}\Delta W) \quad [7]$$

$$\gamma_i = \text{logit}^{-1}\,(\beta_{0i} + \beta_{1i}\Delta P + \beta_{2i}\Delta T + \beta_{3i}\Delta A + \beta_{4i}\Delta U + \beta_{5i}\Delta W) \quad [8]$$

where ΔP is the change in annual precipitation, ΔT is the change in mean annual temperature, ΔA is the change in agricultural land cover, ΔU is the change in urban land cover and ΔW is the change in surface water cover.

For the counterfactual analyses, species-specific coefficients were used with regional averages of climate and land-use change variables (Table S7). These averages are listed in the following equations. Subscript *i* for species is omitted from these equations.

For Los Angeles (LA):

$\psi_{LU, LA}$

$$\phi_{LU, LA} = \text{logit}^{-1}\,(\alpha_0 + \alpha_1{*}0.455 + \alpha_2{*}{-2.471} + \alpha_3{*}{-0.548} + \alpha_4{*}0.531 + \alpha_5{*}0.029) \quad [9]$$

$$\gamma_{LU, LA} = \text{logit}^{-1}\,(\beta_0 + \beta_1{*}0.455 + \beta_2{*}{-2.471} + \beta_3{*}{-0.548} + \beta_4{*}0.531 + \beta_5{*}0.029) \quad [10]$$

$\psi_{CC, LA}$

$$\phi_{CC, LA} = \text{logit}^{-1}\,(\alpha_0 + \alpha_1{*}{-1.028} + \alpha_2{*}1.111 + \alpha_3{*}{-0.154} + \alpha_4{*}{-0.795} + \alpha_5{*}0.441) \quad [11]$$

$$\gamma_{CC, LA} = \text{logit}^{-1}\,(\beta_0 + \beta_1{*}{-1.028} + \beta_2{*}1.111 + \beta_3{*}{-0.154} + \beta_4{*}{-0.795} + \beta_5{*}0.441) \quad [12]$$

For the Central Valley (CV):

$\psi_{LU, CV}$

$$\phi_{LU, CV} = \text{logit}^{-1}\,(\alpha_0 + \alpha_1{*}0.455 + \alpha_2{*}{-2.471} + \alpha_3{*}0.357 + \alpha_4{*}{-0.346} + \alpha_5{*}{-0.029}) \quad [13]$$

$$\gamma_{LU, CV} = \text{logi}^{-1}\text{t}\,(\beta_0 + \beta_1{*}0.455 + \beta_2{*}{-2.471} + \beta_3{*}0.357 + \beta_4{*}{-0.346} + \beta_5{*}{-0.029}) \quad [14]$$

$\psi_{CC, CV}$

$$\phi_{CC, CV} = \text{logit}^{-1}\,(\alpha_0 + \alpha_1{*}0.669 + \alpha_2{*}{-0.724} + \alpha_3{*}{-0.154} + \alpha_4{*}{-0.795} + \alpha_5{*}0.441) \quad [15]$$

$$\gamma_{CC, CV} = \text{logit}^{-1}\,(\beta_0 + \beta_1{*}0.669 + \beta_2{*}{-0.724} + \beta_3{*}{-0.154} + \beta_4{*}{-0.795} + \beta_5{*}0.441) \quad [16]$$

For the Control (CT):

$\boldsymbol{\psi_{CT}}$

ϕ_{CT} = logit^{-1} ($\alpha_0 + \alpha_1$*0.455 + α_2*-2.471 + α_3*-0.154 + α_4*-0.795 + α_5*0.441) [17]

γ_{CT} = logit^{-1} ($\beta_0 + \beta_1$*0.455 + β_2*-2.471 + β_3*-0.154 + β_4*-0.795 + β_5*0.441) [18]

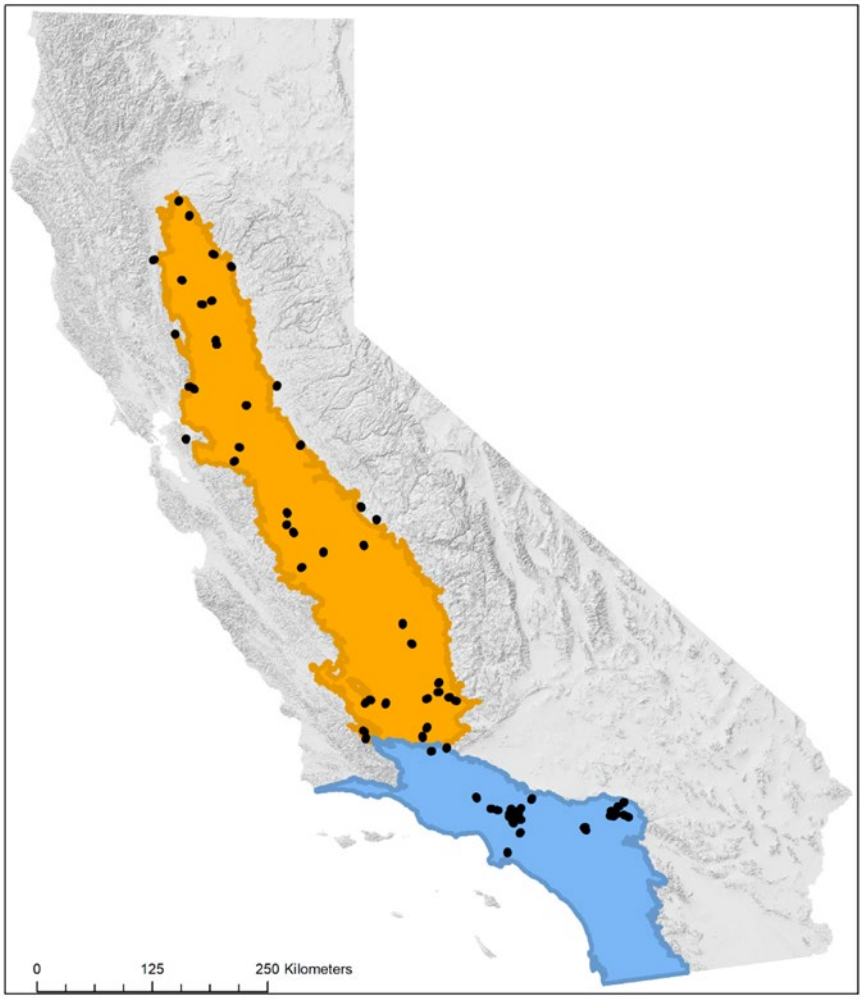

Fig. S1. Map of avian survey locations in California, USA. The orange area indicates the extent of the Great Valley ecoregion that encompasses most of the Central Valley survey sites, and the blue area shows the Southern California Coast and the Southern California Mountains and Valleys ecoregions that encompass the Los Angeles survey sites.

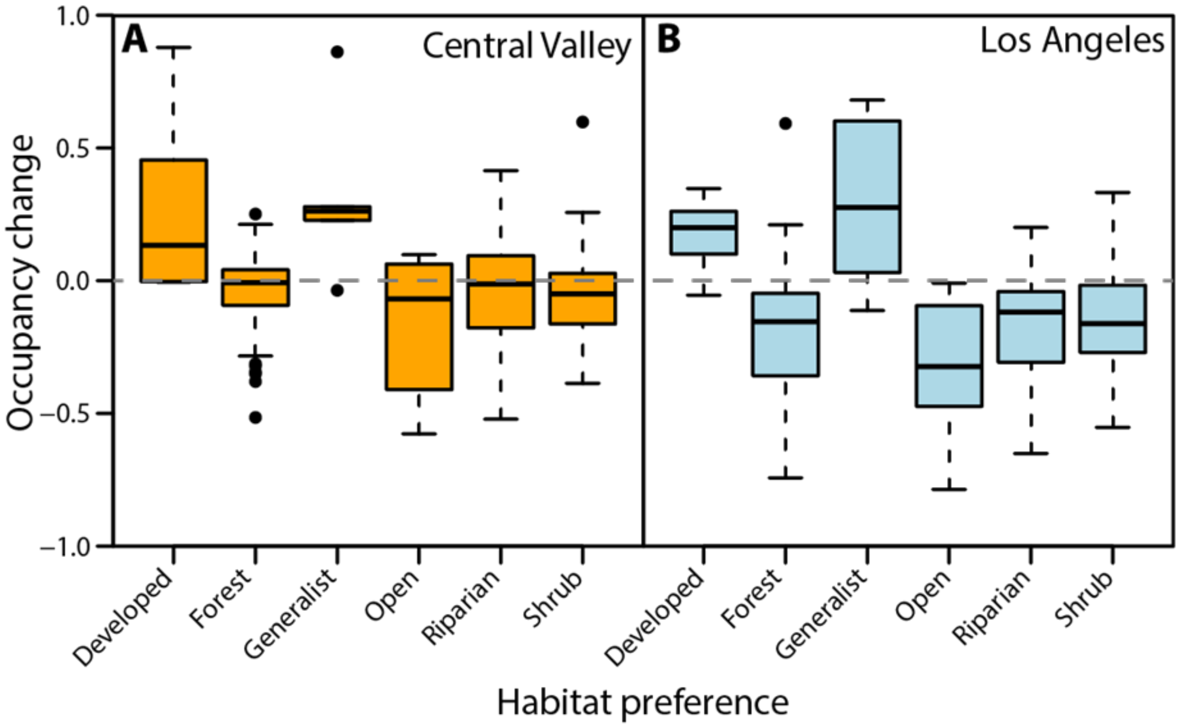

Fig. S2. Occupancy change and habitat preference by study region. Changes in the proportion of sites occupied and habitat preference grouped by region derived from the best model in Table S3.

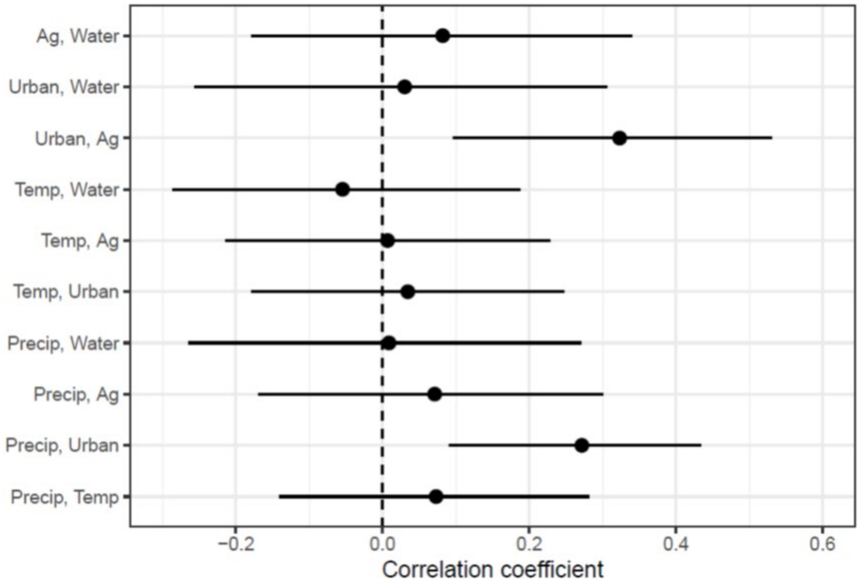

Fig. S3. Mean and 95% credible intervals for correlations among the derivatives of occupancy change for climate and land-use change covariates (see Table S1 for full names) derived from analysis of the full posteriors.

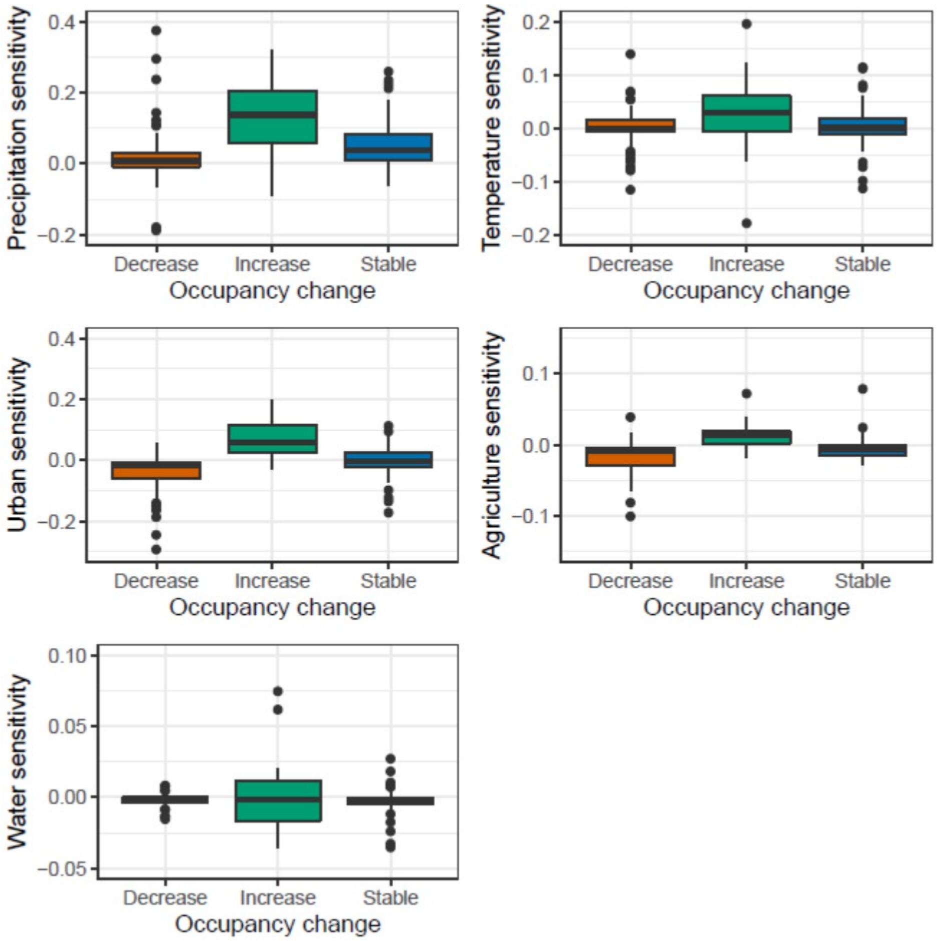

Fig. S4. Occupancy change (significant increase or decrease, or stable indicating not significant) over the past century for 148 bird species in relationship to their sensitivity (derivative of occupancy change) to climate and land-use change. Lower and upper hinges correspond to the first and third quartiles, upper (and lower) whiskers extend from the hinge to the largest (smallest) value no further than 1.5 * the inter-quartile range (distance between the first and third quartiles).

Table S1. Means (± 1 SE) for historic and modern covariates and covariate change over the past century averaged over the survey sites in each region. Climate covariates represent 30-years means corresponding to the historic (1900-1929) and modern (1988-2017) survey periods derived from the PRISM Climate Group (see Methods and Materials). Bolded values indicate significant differences (*P* < 0.05) between modern and historic values within regions using Mann Whitney U-tests.

Region	Covariate	Historic	Modern	Change
Central Valley	Precipitation (mm)	368.72 ± 26.95	379.05 ± 27.77	**11.18 ± 3.23**
	Temperature (°C)	15.87 ± 0.20	16.75 ± 0.20	**0.88 ± 0.03**
	Urban cover (%)	0.04 ± 0.01	0.17 ± 0.03	**0.13 ± 0.02**
	Agriculture cover (%)	0.12 ± 0.03	0.28 ± 0.05	**0.16 ± 0.05**
	Water cover (%)	0.03 ± 0.01	0.02 ± 0.01	-0.02 ± 0.01
	Natural cover (%)	0.81 ± 0.03	0.54 ± 0.06	**-0.27 ± 0.05**
Los Angeles	Precipitation (mm)	621.92 ± 32.43	544.73 ± 27.47	**-77.20 ± 3.89**
	Temperature (°C)	13.63 ± 0.24	15.43 ± 0.72	**1.80 ± 0.04**
	Urban cover (%)	0.05 ± 0.01	0.43 ± 0.07	**0.39 ± 0.02**
	Agriculture cover (%)	0.12 ± 0.05	0.00 ± 0.01	**-0.12 ± 0.06**
	Water cover (%)	0.02 ± 0.01	0.01 ± 0.01	-0.01 ± 0.01
	Natural cover (%)	0.81 ± 0.04	0.56 ± 0.07	**-0.25 ± 0.05**

Table S2. Key species responses discussed in the main text: detected in only one region (CV = Central Valley, LA = Los Angeles), occupancy increased (↑ψ) or declined (↓ψ) by region, top increasing species in both regions (Top ↑ψ), and significant positive (+) or negative (-) covariate relationships.

					Colonization			Persistence		
**Code**	**Species**	**Only in**	**↑ψ CV, ↓ψ LA**	**Top ↑ψ in LA & CV**	**ΔPrecip**	**ΔTemp**	**ΔUrban**	**ΔPrecip**	**ΔUrban**	**ΔAg**
CANG	Canada Goose				+		+			
MALL	Mallard				+		+			
CAQU	California Quail								-	
WITU	Wild Turkey	CV								
RNEP	Ring-necked Pheasant	CV								
INPE	Indian Peafowl	LA								
ROPI	Rock Pigeon			x	+		+			
BTPI	Band-tailed Pigeon					+				
EUCD	Eurasian Collared-Dove			x	+	-				
COGD	Common Ground-Dove	CV								
MODO	Mourning Dove		x		+			+		
ANHU	Anna's Hummingbird			x						
COHU	Costa's Hummingbird								-	
ALHU	Allen's Hummingbird						+			
KILL	Killdeer				+					
GREG	Great Egret				+					
SNEG	Snowy Egret				+					
GRHE	Green Heron				+					
TUVU	Turkey Vulture							+		
RTHA	Red-tailed Hawk								-	
BEKI	Belted Kingfisher							+		
DOWO	Downy Woodpecker				+					

					Colonization				Persistence		
Code	Species	Only in	↑ψ CV, ↓ψ LA	Top ↑ψ in LA & CV	ΔPrecip	ΔTemp	ΔUrban		ΔPrecip	ΔUrban	ΔAg
NUWO	Nuttall's Woodpecker				+						
NOFL	Northern Flicker								-	-	
YCPA	Yellow-crowned Parrot	LA				+	+				
RCPA	Red-crowned Parrot	LA				+	+				
ATFL	Ash-throated Flycatcher									-	-
CAKI	Cassin's Kingbird	LA									
WEKI	Western Kingbird				+				+		
WEWP	Western Wood-Pewee									-	
BLPH	Black Phoebe		x						+	+	
CAVI	Cassin's Vireo									-	
LOSH	Loggerhead Shrike									-	
PIJA	Pinyon Jay	LA									
CLNU	Clark's Nutcracker	LA									
YBMA	Yellow-billed Magpie	CV									
AMCR	American Crow			x	+		+				
OATI	Oak Titmouse					+					
HOLA	Horned Lark									-	
VGSW	Violet-green Swallow				+						
CLSW	Cliff Swallow				+				+		
RWBU	Red-whiskered Bulbul	LA				+	+				
WREN	Wrentit									-	
PHAI	Phainopepla									-	
BRCR	Brown Creeper		x				+				
BGGN	Blue-gray Gnatcatcher									-	
CAGN	California Gnatcatcher	LA									
CANW	Canyon Wren									-	

					Colonization				Persistence		
**Code**	**Species**	**Only in**	**$\uparrow\psi$ CV, $\downarrow\psi$ LA**	**Top $\uparrow\psi$ in LA & CV**	**ΔPrecip**	**ΔTemp**	**ΔUrban**		**ΔPrecip**	**ΔUrban**	**ΔAg**
CACW	Cactus Wren	LA									
HOWR	House Wren		x		+						
LCTH	Le Conte's Thrasher	CV									
NOMO	Northern Mockingbird				+						
EUST	European Starling			x	+		+				
TOSO	Townsend's Solitaire	LA									
HOSP	House Sparrow				+						
HOFI	House Finch								+		
PUFI	Purple Finch									-	
CAFI	Cassin's Finch	LA									
LAGO	Lawrence's Goldfinch									-	
LASP	Lark Sparrow									-	
BCSP	Black-chinned Sparrow									-	
DEJU	Dark-eyed Junco								-		
VESP	Vesper Sparrow	CV									
WEME	Western Meadowlark									-	
BUOR	Bullock's Oriole								+		
TRBL	Tricolored Blackbird	CV									
BHCO	Brown-headed Cowbird			x	+						
BRBL	Brewer's Blackbird		x						+		
OCWA	Orange-crowned Warbler				+						
BTYW	Black-throated Gray Warbler									-	

Table S3. AICc rankings for linear mixed effects models of traits as predictors of occupancy change, weighted by the inverse of the variance in occupancy change. k is the number of parameters in the model.

Model	k	AICc	ΔAICc	AICc weight
Habitat	7	0.50	0	1.00
Tolerance of human habitat modification	3	14.16	13.66	0.00
Migratory behavior	4	54.01	53.51	0.00
Diet category	8	59.74	59.23	0.00
Null	2	68.92	68.42	0.00
Body mass	3	69.07	68.57	0.00

Table S4. WAIC model selection for the spatial scale of land-use change covariates. Models differed only in the scale at which land-use change was measured. pD_{WAIC} is the bias correction term that represents a measure of the model complexity.

Spatial Scale	WAIC	pD_{WAIC}
1000 m	31884.39	1564.89
500 m	31900.76	1544.78
200 m	31955.65	1512.17
100 m	31998.92	1514.77

Table S5. Mean derivatives for occupancy change for 148 bird species in relation to change in mean annual temperature, annual precipitation, urban cover, agricultural cover, and water cover.

Code	Common name	Temp	Precip	Urban	Ag	Water
CANG	Canada Goose	0.0492	0.2146	0.1406	0.0112	-0.0010
MALL	Mallard	0.0184	0.1931	0.1135	0.0208	-0.0111
MOUQ	Mountain Quail	0.0123	0.0155	-0.0576	-0.0101	-0.0021
CAQU	California Quail	-0.0137	0.0394	-0.0241	-0.0016	-0.0036
WITU	Wild Turkey	-0.0145	0.0613	0.0223	0.0063	-0.0117
RNEP	Ring-necked Pheasant	-0.0023	0.0209	0.0042	-0.0017	-0.0019
INPE	Indian Peafowl	0.0102	0.0046	0.0092	0.0006	-0.0001
ROPI	Rock Pigeon	0.0220	0.1958	0.1551	0.0258	0.0617
BTPI	Band-tailed Pigeon	0.1958	-0.0890	0.0099	0.0148	0.0201
EUCD	Eurasian Collared-Dove	-0.1782	0.1947	0.0601	0.0158	0.0203
CGDO	Common Ground-Dove	-0.0159	0.0285	0.0037	0.0027	0.0016
MODO	Mourning Dove	-0.0170	0.0722	0.0245	-0.0025	-0.0014
GRRO	Greater Roadrunner	-0.0177	0.0000	-0.0089	-0.0212	-0.0021
VASW	Vaux's Swift	0.0008	0.1196	0.0375	-0.0058	-0.0239
WTSW	White-throated Swift	0.0268	0.0041	0.0007	-0.0127	-0.0072
BCHU	Black-chinned Hummingbird	-0.0133	0.0940	0.0019	0.0083	0.0089
ANHU	Anna's Hummingbird	0.0695	0.0375	0.0356	0.0021	-0.0088
COHU	Costa's Hummingbird	0.0154	-0.0189	-0.0560	-0.0062	-0.0009
CAHU	Calliope Hummingbird	-0.0126	0.0413	-0.0087	-0.0061	-0.0008
ALHU	Allen's Hummingbird	0.1144	0.0105	0.0947	-0.0194	-0.0047
KILL	Killdeer	-0.0640	0.2122	0.0269	0.0070	0.0007
GBHE	Great Blue Heron	0.0239	0.0371	-0.0333	-0.0126	0.0008
GREG	Great Egret	-0.0159	0.2427	0.0761	0.0177	0.0089
SNEG	Snowy Egret	0.0334	0.1027	0.0248	0.0059	0.0183
GRHE	Green Heron	-0.0152	0.0752	0.0437	0.0031	0.0105
BCNH	Black-crowned Night-Heron	0.0089	0.0839	0.0009	-0.0205	-0.0004
TUVU	Turkey Vulture	-0.0598	0.2950	-0.0664	-0.0810	0.0050
GOEA	Golden Eagle	-0.0207	0.0741	-0.0414	-0.0075	-0.0021
NOHA	Northern Harrier	-0.0060	0.0268	-0.0218	-0.0048	-0.0014
SSHA	Sharp-shinned Hawk	-0.0116	0.0832	-0.0217	-0.0073	-0.0022
COHA	Cooper's Hawk	0.0758	0.0117	0.0799	-0.0270	0.0074
RSHA	Red-shouldered Hawk	0.0032	0.1381	0.0774	-0.0177	-0.0352
SWHA	Swainson's Hawk	0.0380	0.0839	-0.0998	0.0393	-0.0049
RTHA	Red-tailed Hawk	0.0179	0.0895	-0.1233	-0.0172	-0.0089
BUOW	Burrowing Owl	0.0037	0.0009	-0.0045	-0.0031	-0.0005
BEKI	Belted Kingfisher	-0.0202	0.1389	-0.0181	-0.0105	0.0003
LEWO	Lewis's Woodpecker	0.0010	0.0039	-0.0057	-0.0016	-0.0003
ACWO	Acorn Woodpecker	0.0097	0.0253	0.0041	-0.0081	-0.0018

RBSA	Red-breasted Sapsucker	0.0102	-0.0086	-0.0148	-0.0046	-0.0013
DOWO	Downy Woodpecker	-0.0162	0.1231	0.0136	0.0031	0.0033
NUWO	Nuttall's Woodpecker	0.0205	0.1238	0.0516	-0.0069	-0.0300
HAWO	Hairy Woodpecker	-0.0036	-0.0224	-0.0574	-0.0074	-0.0025
WHWO	White-headed Woodpecker	0.0067	-0.0082	-0.0082	-0.0031	-0.0005
NOFL	Northern Flicker	-0.0256	-0.1783	-0.2928	-0.0032	-0.0025
AMKE	American Kestrel	0.0033	0.0437	0.0539	-0.0343	0.0002
YCPA	Yellow-crowned Parrot	0.0401	0.0153	0.0231	0.0006	-0.0013
RCPA	Red-crowned Parrot	0.0526	0.0181	0.0259	0.0030	0.0018
ATFL	Ash-throated Flycatcher	0.0158	0.0434	-0.1641	-0.1003	-0.0034
CAKI	Cassin's Kingbird	-0.0013	0.0091	-0.0017	-0.0020	-0.0002
WEKI	Western Kingbird	-0.1154	0.2372	-0.0401	-0.0058	-0.0083
OSFL	Olive-sided Flycatcher	-0.0314	0.0157	-0.0519	-0.0133	-0.0020
WEWP	Western Wood-Pewee	-0.0427	-0.0608	-0.1352	-0.0118	-0.0032
WIFL	Willow Flycatcher	-0.0004	0.0171	-0.0217	-0.0043	-0.0004
HAFL	Hammond's Flycatcher	-0.0012	0.0083	-0.0074	-0.0025	-0.0003
GRFL	Gray Flycatcher	-0.0084	0.0292	-0.0193	-0.0035	-0.0006
PSFL	Pacific-slope Flycatcher	0.0581	-0.0389	-0.0057	-0.0161	-0.0024
BLPH	Black Phoebe	0.0570	0.2349	0.1128	-0.0058	-0.0091
SAPH	Say's Phoebe	-0.0336	-0.0166	0.0075	-0.0143	-0.0015
BEVI	Bell's Vireo	0.0008	-0.0044	0.0051	-0.0060	-0.0003
HUVI	Hutton's Vireo	0.0079	0.0130	-0.0291	-0.0053	-0.0010
CAVI	Cassin's Vireo	-0.0034	0.0181	-0.0558	-0.0086	-0.0014
WAVI	Warbling Vireo	0.0305	0.0655	-0.0088	-0.0237	-0.0055
LOSH	Loggerhead Shrike	-0.0432	-0.0586	-0.1405	-0.0422	-0.0079
PIJA	Pinyon Jay	0.0002	0.0043	-0.0040	-0.0016	-0.0004
STJA	Steller's Jay	0.0063	-0.0114	-0.0398	-0.0047	-0.0003
CASJ	California Scrub-Jay	0.0422	0.0930	0.0580	0.0055	-0.0300
CLNU	Clark's Nutcracker	0.0010	-0.0003	-0.0065	-0.0027	-0.0005
YBMA	Yellow-billed Magpie	0.0008	0.0386	-0.0036	-0.0007	-0.0004
AMCR	American Crow	0.1215	0.2388	0.1982	0.0724	0.0070
CORA	Common Raven	0.0609	-0.0257	0.0312	-0.0027	-0.0010
MOCH	Mountain Chickadee	-0.0014	-0.0079	-0.0140	-0.0052	-0.0001
OATI	Oak Titmouse	0.1006	0.0463	-0.0272	0.0132	-0.0145
HOLA	Horned Lark	-0.0483	-0.0011	-0.1236	0.0159	0.0018
TRES	Tree Swallow	0.0004	0.0872	-0.0226	-0.0091	0.0044
VGSW	Violet-green Swallow	-0.0566	0.0569	-0.0455	-0.0245	0.0079
NRWS	Northern Rough-winged Swallow	0.0088	0.0333	0.0236	0.0168	-0.0056
PUMA	Purple Martin	0.0003	0.0085	-0.0019	-0.0023	-0.0005
BARS	Barn Swallow	0.0227	0.0816	-0.0031	-0.0137	0.0031
CLSW	Cliff Swallow	-0.0985	0.2595	0.0319	0.0058	0.0059
BUSH	Bushtit	0.0091	0.0511	0.0491	-0.0156	-0.0062
RWBU	Red-whiskered Bulbul	0.0195	0.0120	0.0114	-0.0003	0.0002

WREN	Wrentit	0.0271	0.0005	-0.1242	-0.0384	-0.0052
RCKI	Ruby-crowned Kinglet	0.0185	-0.0275	-0.0248	-0.0160	-0.0008
PHAI	Phainopepla	0.0542	-0.0645	-0.1096	-0.0479	-0.0086
RBNU	Red-breasted Nuthatch	-0.0004	0.0066	-0.0054	-0.0005	-0.0002
WBNU	White-breasted Nuthatch	-0.0249	0.0046	-0.0001	-0.0136	-0.0063
PYNU	Pygmy Nuthatch	-0.0047	0.0061	-0.0073	-0.0062	0.0005
BRCR	Brown Creeper	-0.0078	0.0253	-0.0010	-0.0036	-0.0007
BGGN	Blue-gray Gnatcatcher	0.0669	-0.0011	-0.1231	-0.0466	-0.0073
CAGN	California Gnatcatcher	-0.0003	0.0043	-0.0053	-0.0006	-0.0002
ROWR	Rock Wren	-0.0074	0.0159	-0.0313	-0.0078	-0.0018
CANW	Canyon Wren	0.0318	-0.0300	-0.0261	-0.0066	-0.0015
CACW	Cactus Wren	0.0000	0.0035	-0.0033	-0.0006	-0.0001
BEWR	Bewick's Wren	0.0523	0.0337	0.0700	-0.0206	-0.0108
HOWR	House Wren	0.1119	0.1794	-0.0983	-0.0158	-0.0352
MAWR	Marsh Wren	0.0012	0.0170	-0.0092	-0.0019	-0.0005
CATH	California Thrasher	0.0227	-0.0448	-0.0380	-0.0446	-0.0057
LCTH	Le Conte's Thrasher	0.0019	0.0017	-0.0016	-0.0014	-0.0002
NOMO	Northern Mockingbird	0.1158	0.3202	0.1195	0.0146	-0.0187
EUST	European Starling	-0.0578	0.2516	0.1206	0.0341	0.0145
WEBL	Western Bluebird	-0.0179	-0.0016	-0.0217	-0.0256	-0.0081
MOBL	Mountain Bluebird	0.0001	0.0052	-0.0023	-0.0014	-0.0003
TOSO	Townsend's Solitaire	-0.0008	0.0117	-0.0096	-0.0041	-0.0007
SWTH	Swainson's Thrush	0.0035	0.0181	-0.0177	-0.0119	-0.0011
HETH	Hermit Thrush	-0.0001	0.0098	-0.0066	-0.0035	-0.0003
AMRO	American Robin	-0.0726	0.0643	0.0022	0.0789	-0.0175
HOSP	House Sparrow	-0.0617	0.1629	0.0967	0.0396	0.0746
HOFI	House Finch	-0.0073	0.0471	0.0210	-0.0039	-0.0012
PUFI	Purple Finch	0.0020	-0.0317	-0.1050	-0.0192	-0.0035
CAFI	Cassin's Finch	0.0006	0.0041	-0.0076	-0.0025	-0.0006
PISI	Pine Siskin	0.0006	0.0046	-0.0088	-0.0031	-0.0006
LEGO	Lesser Goldfinch	0.0616	0.0321	0.0347	-0.0264	0.0013
LAGO	Lawrence's Goldfinch	0.0289	0.1133	-0.1718	0.0067	0.0272
AMGO	American Goldfinch	0.0357	0.1068	-0.0210	-0.0277	-0.0132
LASP	Lark Sparrow	-0.0793	0.0212	-0.2450	-0.0272	-0.0035
CHSP	Chipping Sparrow	-0.0322	-0.0156	-0.0373	-0.0165	0.0024
BCSP	Black-chinned Sparrow	-0.0042	-0.0310	-0.0743	-0.0083	-0.0022
BRSP	Brewer's Sparrow	-0.0044	0.0178	-0.0097	-0.0069	-0.0005
DEJU	Dark-eyed Junco	0.0370	-0.1877	-0.0166	-0.0552	0.0003
BESP	Bell's Sparrow	-0.0019	0.0115	-0.0017	-0.0039	-0.0005
VESP	Vesper Sparrow	0.0014	0.0028	-0.0020	-0.0013	-0.0003
SAVS	Savannah Sparrow	0.0177	0.0337	-0.0433	-0.0072	0.0047
SOSP	Song Sparrow	0.0689	0.1107	0.0200	-0.0342	0.0028
CALT	California Towhee	0.0156	0.0825	0.0590	-0.0096	-0.0101

RCSP	Rufous-crowned Sparrow	0.0002	0.0389	-0.0541	-0.0116	-0.0035
GTTO	Green-tailed Towhee	0.0079	-0.0160	-0.0085	-0.0042	-0.0003
SPTO	Spotted Towhee	0.0059	0.0041	0.0145	-0.0040	-0.0066
YBCH	Yellow-breasted Chat	0.0019	0.0105	-0.0073	-0.0094	-0.0004
YHBL	Yellow-headed Blackbird	0.0051	0.0124	-0.0013	-0.0081	-0.0007
WEME	Western Meadowlark	-0.0721	0.0462	-0.1866	-0.0291	0.0083
HOOR	Hooded Oriole	0.0106	0.0213	0.0376	-0.0110	-0.0004
BUOR	Bullock's Oriole	-0.0624	0.3750	-0.1504	-0.0642	-0.0063
RWBL	Red-winged Blackbird	-0.0207	0.1051	-0.0586	0.0022	0.0075
TRBL	Tricolored Blackbird	-0.0293	0.0632	-0.0412	-0.0058	-0.0028
BHCO	Brown-headed Cowbird	0.0283	0.1116	-0.0127	0.0126	-0.0299
BRBL	Brewer's Blackbird	-0.1129	0.2233	-0.0014	-0.0052	-0.0013
GTGR	Great-tailed Grackle	0.0128	0.0789	0.0782	0.0245	-0.0022
OCWA	Orange-crowned Warbler	0.0276	0.1207	-0.0036	-0.0178	-0.0046
NAWA	Nashville Warbler	-0.0048	0.0917	0.0324	-0.0149	0.0006
MGWA	MacGillivray's Warbler	0.0130	-0.0047	-0.0735	-0.0149	-0.0019
COYE	Common Yellowthroat	0.0405	0.0785	-0.0066	-0.0428	-0.0135
YEWA	Yellow Warbler	0.1392	0.1438	0.0463	-0.0570	-0.0153
BTYW	Black-throated Gray Warbler	-0.0039	0.0055	-0.1268	-0.0172	-0.0019
WIWA	Wilson's Warbler	0.0811	0.1764	0.0235	-0.0146	-0.0326
WETA	Western Tanager	0.0095	0.0580	-0.0226	-0.0148	-0.0110
BHGR	Black-headed Grosbeak	0.0394	0.0092	-0.0544	-0.0428	-0.0076
BLGR	Blue Grosbeak	0.0017	0.0136	-0.0179	-0.0282	-0.0055
LAZB	Lazuli Bunting	0.0540	-0.0422	-0.0630	-0.0362	-0.0014

Table S6. Scores and P-values for five phylogenetic signal tests for sensitivity (derivatives of occupancy change) and impacts of climate and land-use change covariates. Tests are Moran's I index (I), Abouheif's C_{mean} index, Blomberg's K, K* and Pagel's λ. The first two tests indicate autocorrelation as a measure of phylogenetic signal. The last three are based on evolutionary models. Significant effects (*P* < 0.05) are bolded.

Analysis	Tree	Factor	C_{mean}	$P(C_{mean})$	I	*P*(I)	K	*P*(K)	K*	*P*(K*)	λ	*P*(λ)
Sensitivity	Average	Temp	-0.022	0.635	-0.004	0.316	0.135	0.562	0.174	0.524	0.000	1.000
		Precip	0.081	0.070	0.000	0.211	**0.207**	**0.008**	**0.234**	**0.023**	0.000	1.000
		Ag	0.018	0.343	-0.002	0.239	0.171	0.144	0.219	0.134	0.000	1.000
		Urban	0.015	0.317	-0.010	0.681	0.132	0.565	0.145	0.841	0.000	1.000
		Water	**0.094**	**0.035**	0.000	0.166	0.185	0.139	0.237	0.098	0.255	0.392
	Majority	Temp	-0.020	0.619	-0.020	0.678	0.018	0.727	0.022	0.660	0.000	1.000
		Precip	0.081	0.058	0.026	0.139	0.023	0.088	0.027	0.076	0.000	1.000
		Ag	0.017	0.309	0.011	0.235	0.022	0.187	0.027	0.171	0.000	1.000
		Urban	0.015	0.328	0.006	0.291	0.024	0.056	0.026	0.159	0.000	1.000
		Water	**0.095**	**0.026**	**0.065**	**0.010**	**0.029**	**0.017**	**0.034**	**0.015**	0.224	0.102
Impacts	Average	LA climate change	0.001	0.441	-0.007	0.496	0.160	0.179	0.194	0.222	0.000	1.000
		LA land use	-0.002	0.472	-0.014	0.831	0.134	0.552	0.148	0.810	0.000	1.000
		CV climate change	0.072	0.079	0.003	0.114	0.126	0.664	0.162	0.653	0.000	1.000
		CV land use	0.048	0.147	0.003	0.117	**0.204**	**0.024**	**0.244**	**0.029**	0.047	0.510
	Majority	LA climate change	0.002	0.410	-0.005	0.446	0.021	0.223	0.025	0.221	0.000	1.000
		LA land use	-0.001	0.447	0.002	0.370	0.022	0.126	0.025	0.333	0.000	1.000
		CV climate change	0.073	0.070	0.023	0.151	0.022	0.170	0.026	0.212	0.000	1.000
		CV land use	0.048	0.159	0.019	0.147	**0.027**	**0.027**	**0.032**	**0.014**	0.026	0.664

Table S7. Covariate change and z scores by region (LA = Los Angeles; CV = Central Valley).

Covariate	Mean change over all sites	SD of change among sites		LA sites mean change	LA sites mean z-score		CV sites mean change	CV sites mean z-score		z-score equivalent to no change
Δ Temperature	1.243	0.503		1.802	1.111		0.879	-0.724		-2.471
Δ Precipitation	-23.671	52.070		-77.195	-1.028		11.182	0.669		0.455
Δ Agriculture	0.047	0.305		-0.120	-0.548		0.156	0.357		-0.154
Δ Urbanization	0.232	0.292		0.387	0.531		0.131	-0.346		-0.795
Δ Water	-0.015	0.034		-0.014	0.029		-0.016	-0.029		0.441

Data S1. (separate file)
Mean and 95% credible intervals for species-specific probabilities of: (1) probability summary (initial occupancy, occupancy change, colonization, persistence and detection); (2) colonization covariate coefficients; (3) survival (persistence) covariate coefficients; (4) initial occupancy covariate coefficients; (5) detection covariate coefficients; and (6) occupancy by region change in occupancy, persistence, colonization and detection.

Data S2. (separate file)
Traits for species in analysis: log-transformed body mass, body length, wingspan, migratory behavior, territorial, altricial (vs prococial), mating behavior, breeding behavior, maximum lifespan, number of broods per year, mean clutch size, nest type, diet, foraging location, nest location, main habitat, human adapter. See Table S1 for guide to species 4-letter codes.
